# Native *Wolbachia* infection and larval competition stress shape fitness and West Nile virus infection in *Culex quinquefasciatus* mosquitoes

**DOI:** 10.3389/fmicb.2023.1138476

**Published:** 2023-03-15

**Authors:** Abdullah A. Alomar, Daniel W. Pérez-Ramos, Dongmin Kim, Natalie L. Kendziorski, Bradley H. Eastmond, Barry W. Alto, Eric P. Caragata

**Affiliations:** Florida Medical Entomology Laboratory, Department of Entomology and Nematology, Institute of Food and Agricultural Sciences, University of Florida, Vero Beach, FL, United States

**Keywords:** *Wolbachia*, *Culex quinquefasciatus*, mosquito, fitness, larval competition, West Nile virus

## Abstract

**Introduction:**

*Wolbachia* transinfections established in key mosquito vectors, including *Aedes aegypti* are typically associated with pathogen blocking—reduced susceptibility to infection with key pathogens and reduced likelihood those pathogens are transmitted to new hosts. Host-symbiont-virus interactions are less well understood in mosquitoes like *Culex quinquefasciatus*, which naturally harbor *Wolbachia*, with pathogen blocking observed in some populations but not others, potentially due to innate differences in their *Wolbachia* load. In nature, mosquito larvae are often subject to developmental stresses associated with larval competition, which can lead to reduced body size and differential susceptibility to arbovirus infection.

**Methods:**

In this study, we sought to understand whether competition stress and *Wolbachia* infection in *Cx. quinquefasciatus* combine to impact host fitness and susceptibility to infection with West Nile virus. We reared *Wolbachia*-infected and uninfected *Cx. quinquefasciatus* larvae under three competition stress levels, increasing larval density without increasing the amount of food supplied. We then monitored larval development and survival, measured wing length and quantified *Wolbachia* density in adults, and then challenged mosquitoes from each treatment group orally with West Nile virus.

**Results and Discussion:**

We observed that high competition stress extended development time, decreased the likelihood of eclosion, decreased body size, and increased susceptibility to West Nile virus (WNV) infection. We also observed that *Wolbachia* infection reduced WNV load under low competition stress, and significantly improved the rate of survival for larval reared under higher competition stress. Consequently, our data suggest that native *Wolbachia* infection in *Cx. quinquefasciatus* has differential consequences for host fitness and susceptibility to WNV infection depending on competition stress.

## 1. Introduction

West Nile virus (WNV) is a neurotropic flavivirus that causes significant and sometimes severe disease in humans. This virus naturally circulates in an enzootic cycle among *Culex* mosquitoes and birds, which are considered an amplifying host for WNV. Members of the *Culex pipiens* complex, including *Cx. pipiens* and *Cx. quinquefasciatus*, are implicated as primary vectors for WNV (Ciota et al., 2013). Since no specific antiviral treatment or licensed vaccine is available for WNV, mosquito control remains the primary strategy used to reduce the incidence of virus transmission (Ronca et al., 2021).

There are several innovative methods for controlling mosquitoes and the pathogens they transmit that utilize the obligate intracellular, endosymbiont bacterium *Wolbachia pipientis*. These maternally inherited bacteria are known for their ability to manipulate host reproductive biology, most notably *via* cytoplasmic incompatibility (CI). The CI phenotype occurs when *Wolbachia*-uninfected females produce inviable embryos after fertilization by *Wolbachia*-infected male sperm (Sicard et al., 2019). CI forms the basis of the incompatible insect technique, where the mass release of *Wolbachia*-infected male mosquitoes leads to the suppression of a target mosquito population. This approach has been successfully applied against mosquito populations in nature in multiple countries (Mains et al., 2019; Zheng et al., 2019; Caputo et al., 2020; Beebe et al., 2021).

*Wolbachia* infection can also induce pathogen blocking, a phenotype characterized by a reduction in the rate of infection, replication, and transmission of key pathogens. This phenotype is common among transinfected mosquitoes, where a stable and heritable *Wolbachia* infection has been established after embryonic microinjection of mosquito eggs (Caragata et al., 2021). Several *Wolbachia* transinfections have been established in *Ae. aegypti* with notable examples including the *w*Mel and *w*AlbB *Wolbachia* strains. These transinfected *Ae. aegypti* lines display strong pathogen blocking against numerous medically important viruses including dengue virus (DENV), chikungunya virus (CHIKV), and Zika virus (ZIKV; Moreira et al., 2009; Bian et al., 2010; Walker et al., 2011; Blagrove et al., 2012, 2013; Van den Hurk et al., 2012; Caragata et al., 2016; Dutra et al., 2016). Several *Wolbachia* transinfections have been developed in *Cx. quinquefasciatus* using strains native to *Ae. albopictus* (Ant et al., 2020), but it is unclear whether they induce pathogen blocking against WNV.

*Wolbachia*-mediated population replacement is a mosquito control strategy that involves the mass release of male and female *Wolbachia*-infected mosquitoes and requires both CI and pathogen blocking. CI is used to drive *Wolbachia* into a mosquito population, and after the *Wolbachia* infection reaches high prevalence within the target population, pathogen blocking limits potential arbovirus transmission. Successful examples of *Wolbachia* population replacement have occurred in multiple countries, and *Wolbachia* infection rates typically remain stable after an initial release period (Nazni et al., 2019; Ryan et al., 2019; Gesto et al., 2021). Critically, the presence of *Wolbachia*-infected mosquitoes can significantly reduce the incidence of dengue in local human populations (Indriani et al., 2020; Ahmad et al., 2021; Pinto et al., 2021; Utarini et al., 2021).

Many mosquito species are naturally infected by *Wolbachia*, including major vectors like those from the *Cx. pipiens* complex and *Ae. albopictus* (Hertig, 1936; Kittayapong et al., 2002; Dumas et al., 2013; Bergman and Hesson, 2021). Transinfections and native *Wolbachia* infections differ in several key parameters. The length of association for transinfections is much shorter, while native-host-symbiont associations might have persisted for tens of thousands of years (Caragata et al., 2017). Native host-symbiont interactions are characterized by tolerance, potentially due to a lengthy period of co-adaptation (Zug and Hammerstein, 2015a, 2015b). In contrast, host-symbiont relationships in transinfections are more likely to demonstrate resistance on the part of the host leading to large-scale transcriptional dysregulation, particularly of genes involved in immunity and response to stress (Pan et al., 2012; Rancès et al., 2012). Host fitness costs in transinfections are typically moderate to high, with stronger fitness costs potentially associated with higher bacterial density *Wolbachia* strains like *w*MelPop (McMeniman et al., 2009) while moderate density strains such as *w*Mel induce only minor fitness effects. Native *Wolbachia* infections are typically characterized by lower *Wolbachia* density than transinfections (Moreira et al., 2009), but they can still alter host fitness and molecular biology (Caragata et al., 2017; Nascimento da Silva et al., 2022). For instance, in *Ae. albopictus,* native *Wolbachia* infection enhances female longevity, fecundity, and eggs hatch rates relative to uninfected females (Dobson, 2004), while in *Cx. quinquefasciatus*, native *Wolbachia* infection reduces host fecundity and fertility (de Almeida et al., 2011).

The extent to which native *Wolbachia* infections modulate host-virus interactions and virus transmission in mosquito populations in nature remains unclear. This is important as native *Wolbachia* infections are highly prevalent in mosquito populations that are directly responsible for transmitting important pathogens. Pathogen blocking does occur in insects with native *Wolbachia* infections, with that phenotype first observed in *Drosophila melanogaster* (Hedges et al., 2008; Teixeira et al., 2008). However, in mosquitoes, interactions between hosts, pathogens, and native *Wolbachia* infections do not always lead to pathogen blocking, and when it does occur it is generally weaker than what is seen with transinfections. For instance, in *Ae. albopictus* from La Reunion Island, removal of *Wolbachia* modulated CHIKV infection (Mousson et al., 2010), and modestly increased the likelihood of DENV transmission (Mousson et al., 2012). However, no impact on CHIKV infection was observed in a similar study on *Ae. albopictus* from Malaysia (Ahmad et al., 2017). Interestingly, the presence of the *w*AlbB strain in the C6/36 mosquito cell line severely limits replication of Flaviviruses (DENV, ZIKV, and WNV) and Alphaviruses (Ross River, Barmah Forest, and Sindbis; Ekwudu et al., 2020).

Native *Wolbachia* infection in *Cx. quinquefasciatus* mosquitoes has been linked to lower WNV titers and decreased transmission rates (Glaser and Meola, 2010), although any pathogen-blocking effects in both *Cx. quinquefasciatus* and *Cx. pipiens* might be limited to specific populations of mosquitoes (Micieli and Glaser, 2014). The strength of pathogen blocking in any host-symbiont combination has been linked to *Wolbachia* density for both native (Micieli and Glaser, 2014) and transinfections (Walker et al., 2011; Joubert et al., 2016). Both *Wolbachia* density and *Wolbachia*-host interactions can be strongly modulated by extrinsic factors including temperature (Hurst et al., 2000; Yi et al., 2016; Hague et al., 2020; Lau et al., 2020) and nutrient availability (Dutton and Sinkins, 2004; Caragata et al., 2014; Ponton et al., 2015). Accordingly, variation in these factors could feasibly be expected to impact many aspects of the *Wolbachia*-host relationship, including host-pathogen-symbiont tripartite interactions.

In nature, mosquitoes are subjected to many different abiotic and biotic stressors that can affect their biology and their interactions with pathogens. Larval competition for limited resources and space are common biotic stresses that detrimentally affect many fitness-linked traits, including development time, adult survival, and adult size (Juliano and Philip Lounibos, 2005; Juliano, 2007). Crowded larval conditions can extend development time and produce smaller adults in *Ae. aegypti* and *Ae. albopictus* (Noden et al., 2016). Critically, smaller adults resulting from competition stress can exhibit enhanced vector competence of pathogens, including DENV and Sindbis virus (Alto et al., 2005, 2008). Larval competition can also alter *Wolbachia*-host dynamics, with consequences for host fitness. For example, under high competition stress, *w*MelPop-transinfected *Ae. aegypti* experienced prolonged development time and decreased adult size comparing to uninfected mosquitoes (Ross et al., 2014). Similar effects occur for native *Wolbachia* infections. In *Ae. albopictus*, *Wolbachia* infection extends larval development time and reduces the adult eclosion rate, but only under high competition conditions (Gavotte et al., 2010, 2014). Likewise, *Wolbachia* density in *Ae. albopictus* is reduced during larval crowding and nutritional stress (Dutton and Sinkins, 2004).

The impact of competition stress on pathogen blocking in mosquitoes with a native *Wolbachia* infection is not well characterized. To that end, we sought to improve understanding of the role of larval ecology as a modulator of host-symbiont-pathogen tripartite interactions in mosquitoes with a native *Wolbachia* infection. Utilizing *Cx. quinquefasciatus* and WNV as a model system, we examined the impact of varying larval competition stress and the presence or absence of the native *w*Pip *Wolbachia* infection on mosquito development, fitness, *Wolbachia* density, and WNV infection.

## 2. Materials and methods

### 2.1. Biosafety information

All WNV experiments were conducted in a Biosafety Level (BSL-3) and Arthropod Containment Level (ACL-3) facility. For respiratory protection, all personnel wore powered air purifying respirators (3 M Versaflo Healthcare PAPR TR-600-HKL) or N95 respirators. All animal and virus work was conducted in accordance with protocols approved by the University of Florida’s Institutional Biosafety Committee and Institutional Animal Care and Use Committee.

### 2.2. Mosquito rearing

The *Cx. quinquefasciatus* mosquitoes used in this experiment were originally collected as larvae from a small pool located near the Florida Medical Entomology Laboratory, Vero Beach, during 2019 and were maintained under standard insectary conditions for 18 generations prior to this project. Through quantitative qPCR using primers for the *w*Pip phage WO (*orf7*-F:GTTTGTGCAGCTAATAG; *orf7*-R: GTCTGCA AGGCCTATTTCTACTG; Zheng et al., 2019; protocol described below), this line was determined to be infected by *Wolbachia*. Colony larvae were fed an equal mixture of dried *Saccharomyces cerevisiae* yeast and lactalbumin (150 mg) every 4 days until pupation. Pupae were collected daily from each tray and transferred to plastic cups containing distilled water and placed inside cages (Volume = 0.027 m^3^). Newly emerged adults were given 10% sucrose solution *ad libitum* through cotton pledgets. Both colony and experimental mosquitoes were maintained in a climate-controlled walk-in incubator at 26 ± 2°C and 60 ± 10% relative humidity with a 12 h light/dark cycle, according to standard rearing procedures.

### 2.3. Generation of the *Wolbachia*-free line

The experiments described in this study utilized two *Cx. quinquefasciatus* colonies, a wild-type colony naturally infected by *Wolbachia* (WT), and a second line derived from the WT colony where the *Wolbachia* infection was removed by treatment with the antibiotic tetracycline hydrochloride (Tet). Adult WT mosquitoes were fed on 1 mg/mL antibiotic tetracycline hydrochloride (Tet; Catalog No. AAB2140814, Thermo Fisher Scientific, Waltham, MA, United States) dissolved in 10% sucrose solution for three consecutive generations. The elimination of *Wolbachia* was confirmed by qPCR. *Wolbachia* infection was detected by qPCR and 2x SsoAdvanced universal SYBR green supermix (Bio-Rad, United States), using *Wolbachia* 16S ribosomal RNA gene (16S rRNA-F: GAGTGA AGAAGG CCTTTGGG; 16S rRNA-R: CACGGAGTTAGCCA GGACTTC; Fraser et al., 2020) with the following program: 95°C for 5 min, then 35 cycles of 95°C for 5 s, and 60°C for 30 s. After the successful *Wolbachia* elimination was confirmed, water was collected from WT colony rearing trays, post-pupation. For two consecutive generations prior to the commencement of experiments described below, 1 mL aliquots of this water were added to Tet colony larval rearing trays containing second instar larvae, in order to normalize the environmental microbiota between the two colonies.

### 2.4. Larval competition manipulation

In these experiments, larval competition stresses consisted of both crowding stress (varied larval numbers within a defined volume of water) and nutritional stress (food availability per larva). Eggs from both lines were hatched synchronously under vacuum for 45 min. Newly hatched (≤ 24 h-old) first-instar larvae from WT and Tet lines were subjected to three intraspecific larval competition levels, consisting of 100 (low competition stress), 200 (medium competition stress), and 300 (high competition stress) larvae in 2 L of distilled water in plastic rearing trays. Our six experimental treatments are referred to as follows in the text: (WT-100; Tet-100; WT-200; Tet-200; WT-300; and Tet-300). Five trays of larvae for each treatment were prepared for a total of 30 experimental units. Each larval tray was provided with 150 mg of larval diet (1:1 *Saccharomyces cerevisiae* yeast and lactalbumin), with an additional 150 mg of food provided 4 days later. After this, no further food was provided. Pupae were collected daily from each tray and transferred to plastic cups containing distilled water and placed inside cages (0.027 m^3^). Adults from each treatment were maintained independently and were provided with 10% sucrose solution *ad libitum*. For each experimental tray, we recorded the development time (A. time from hatching to pupation, B. time to male eclosion, and C. time to female eclosion), and the adult eclosion rate (proportion of individuals reaching adulthood from the initial number of larvae added), with these data collected daily.

### 2.5. Adult size assay

To estimate the effect of competition and *Wolbachia* infection on adult female mosquito body size, groups of females (*N* = 20) were collected from each competition treatment. Wing length measurements were performed as described previously (Alomar et al., 2020). Briefly, a single wing was dissected from each female, placed on glass microscope slide (Cardinal Health, Dublin, OH, United States), and measured from the alular notch to the wing tip, excluding the wing fringe (Nasci, 1986). Wing length was measured in millimeters using computer imaging software (IMT i-Solution lit, Princeton, NJ, United States) with a phase contrast microscope.

### 2.6. *Wolbachia* density quantification

Genomic DNA was extracted from individual adult, female *Wolbachia*-infected mosquitoes (*N* = 20) collected at 5 days post-eclosion using DNeasy Blood & Tissue Kits (QIAGEN) according to the manufacturer’s instructions. *Wolbachia* was quantified by qPCR and 2x SsoAdvanced universal SYBR green supermix (Bio-Rad, United States). qPCR primers were synthesized by Integrated DNA Technologies (Coralville, IA, United States) to amplify a fragment of the conserved *Wolbachia* 16S ribosomal RNA gene (16S rRNA-F: GAGTGAAGAAGGCCTTTGGG; 16S rRNA-R: CACGGAG TTAGCCAGGACTTC; Fraser et al., 2020) and the mosquito homothorax gene (qHTH-F: TGGTCCTATATTGGCGAGCTA; qHTH-R: TCGTTTTTGCAAGAAGGTCA; Ant et al., 2020). qRT-PCR reactions were performed in duplicate and containing 0.5 μL of each 2.5 μM primer, 5 μL of SYBR green, 2 μL of extracted DNA, and 2 μL of nuclease-free water in a total volume of 10 μL. Total *Wolbachia* density was measured by quantifying the copy number of the 16S rRNA gene relative to the qHTH reference gene. qPCR was performed on a BioRad CFX-96 real-time PCR detection system (Bio-Rad, United States) with the following program: 95°C for 5 min, then 35 cycles of 95°C for 5 s and 60°C for 30 s, followed by a melt-curve analysis (65–95°C with 0.5°C increments, 2–5 s/step). Mean normalized expression values were calculated using Q-Gene. (Simon, 2003).

### 2.7. West Nile virus propagation and experimental oral infection

Kidney epithelial cells (Vero E6) of the African green monkey *Cercopithecus aethiops* (American Type Culture Collection, Manassas, VA, United States) were grown in culture medium 199 (M199; HyClone, GE Healthcare, Logan, UT, United States) supplemented with 10% heat-inactivated fetal bovine serum (Thermo Fisher Scientific, Waltham, MA, United States), penicillin–streptomycin, and mycostatin and maintained in an incubator at 37°C with 5% carbon dioxide. The West Nile virus isolate used in this study (strain FLO3-FL2-3; GenBank accession no. DQ983578.1) was isolated from a pool of *Cx. nigripalpus* from Indian River County, Florida, in 2003 (Alto et al., 2014) and propagated in Vero cells thereafter. To prepare WNV-infected blood meals, confluent monolayers of Vero cells were inoculated with 200 μl of WNV stock [8 log_10_ plaque-forming units per milliliter (PFU/mL)] and incubated for 1 h at 37°C and 5% carbon dioxide to facilitate attachment of the virus to cells, after which 24 mL of M199 media supplemented with 10% heat-inactivated FBS, penicillin–streptomycin, and mycostatin was added, followed by a further incubation for 3 days.

WT and Tet females from each experimental treatment were placed in 16 oz. cardboard cages (50/cage), transferred to the FMEL BSL-3/ACL-3 laboratory, and starved overnight prior to oral feeding on WNV-infected bloods. Three replicate cages were used for each treatment. Mosquitoes used in this assay were aged between 5 and 15 days post-eclosion, with this difference reflecting competition stress treatment-associated differences in development. Mosquitoes were allowed to feed for 1 h on a mixture of anticoagulated chicken blood (Hemostat Laboratories, Dixon, CA, United States) and cell culture supernatant containing freshly harvested WNV virus (1:1 ratio). Bloodmeals were provided using a Hemotek membrane blood-feeding system (Hemotek, Blackburn, United Kingdom) pre-heated to 37°C for 1 h. Aliquots of 1 ml were taken from WNV-containing bloodmeal, placed into 2 ml cryogenic vials (MilliporeSigma, Burlington, MA, United States), and stored at −80°C for later titration. After feeding, mosquitoes were immobilized using carbon dioxide gas for sorting and only fully engorged females were retained. These mosquitoes were placed in new cardboard cages, and offered 10% sucrose solution, which was renewed every 2 days. Cages of mosquitoes were maintained for 14 days post infection (dpi) in a climate-controlled incubator at 26 ± 2°C, 60 ± 10% relative humidity, and in a 12 h light/dark cycle. After this time, mosquitoes were dissected using sterile forceps to remove their legs from bodies and placed in separate microcentrifuge tubes containing 1 mL of M199.

### 2.8. Collection of saliva from WNV-challenged mosquitoes

Mosquito saliva, as a proxy for WNV transmission, was collected from mosquitoes by forced salivation using microhematocrit capillary tubes containing type B immersion oil (Cargille Laboratories, Cedar Grove, NJ, United States). The proboscis of each mosquito was inserted in a capillary tube, then mosquitoes were left to salivate for 1 h. After this time, the contents of each capillary were independently deposited in a microcentrifuge tube containing 300 μL of M199 media. Body samples (thorax+abdomen) from each of these mosquitoes were then dissected and placed in microcentrifuge tubes containing 1 mL of M199 media. All mosquito samples were stored at −80°C until further processing.

### 2.9. Plaque-forming assay

West Nile virus infection, dissemination, and transmission as well as WNV load were determined for each mosquito specimen *via* plaque-forming assays performed using Vero cells as described elsewhere (Alomar et al., 2022). Cells were seeded in 12 well plates at a density of 150,000 cells/well in 200 μL of M199 media supplemented with 10% FBS, penicillin–streptomycin, and mycostatin. Cells were maintained at 37°C with 5% carbon dioxide. The next day, mosquito samples were homogenized using a TissueLyser II sample disruptor (Qiagen, Germantown, MD, United States) at 19.5 Hz for 3 min, then centrifuged at 13,200 rpm for 5 min. 10-fold serial dilutions of each mosquito sample were made and added to individual plate wells (200 μL/well). Plates were then incubated at 37°C and 5% carbon dioxide atmosphere for 1 h. After this time, a 1% immobilizing overlay of methylcellulose was added to each well of the plates (1.5 mL/well), which were then incubated for 3 days. After this, overlays were removed from wells, then plates were stained with 0.25% crystal violet solution (1 mL/well) for 45 min. Stained plates were washed with tap water, dried, and scored for cytopathic effect. Each well was classified as WNV-positive or WNV-negative based on the presence or absence of WNV cytolytic plaques, respectively. Viral loads were calculated across five dilutions per sample (10^−1^–10^−5^), with values expressed as plaque-forming units per milliliter (PFU/mL). Mosquito susceptibility to WNV infection (proportion of WNV-positive body specimens out of the total mosquito body specimens tested), dissemination (proportion of positive leg specimens from mosquitoes with WNV-positive bodies), and transmission (proportion of positive saliva samples from mosquito specimens with WNV-positive legs) were determined based on the presence of infectious WNV particles.

### 2.10. Statistical analysis

*Wolbachia* infection and larval competition effects on WNV infection, dissemination, transmission and adult eclosion were analyzed using logistic regression analysis. Mosquito development time was analyzed using Poisson regression analysis. Two-way ANOVA models were performed to test for the effects of treatments on adult body size (wing length) and WNV loads. Tukey’s *post hoc* tests were used for pairwise comparisons between treatments after detection of significant effects. *Wolbachia* density data were not normally distributed, as determined through Kolmogorov–Smirnov tests. Those data were analyzed using the nonparametric Kruskal-Wallis ANOVA with Dwass-Steel-Critchlow-Fligner pairwise comparisons as *post hoc* tests. Comparisons were not considered statistically significant at *p* values greater than 0.05. Analyses were conducted using SAS statistical software (version 9.4, SAS Institute Inc., Cary, NC). GraphPad Prism 6 (GraphPad Software, Inc.) was used to prepare figures.

## 3. Results

### 3.1. Development time and adult eclosion

**Figure 1:**
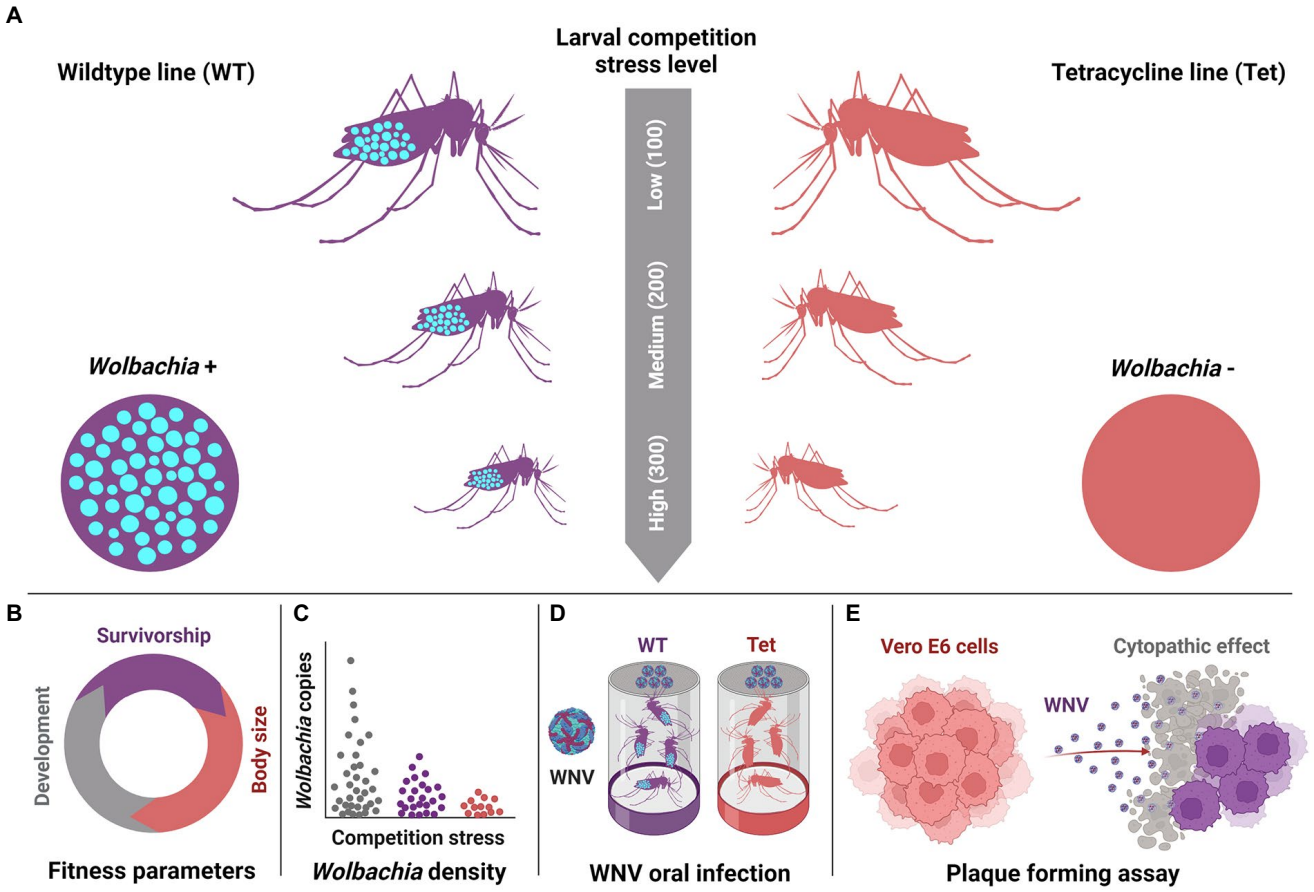
Schematic overview of experimental design. WT (*Wolbachia*+, purple) and Tet (*Wolbachia-*, red) lines of *Culex quinquefasciatus* mosquitoes were subject to three levels of larval competition stress (low, medium, high), with higher competition producing adult mosquitoes with smaller body size **(A)**. Mosquito fitness (larval development time, pupation, and adult eclosion rates) were determined for mosquitoes from each experimental treatment **(B)**. Genomic DNA was isolated from whole mosquitoes (collected at 5 days post-eclosion) and used in a SYBR green-based qPCR assay to compare *Wolbachia* density across the three WT treatments **(C)**. Adult female mosquitoes from each of the six treatments were orally challenged with WNV (strain FLO3-FL2-3) *via* oral infection to determine whether competition and/or *Wolbachia* infection altered the course of infection **(D)**. WNV infection in mosquito tissues (bodies, legs, and saliva all at 14 days post-infection) was evaluated *via* plaque-forming assay to assess the impact of *Wolbachia* infection and competition stress on mosquito susceptibility to WNV infection, and rates of WNV dissemination and transmission **(E)**.

**Figure 2:**
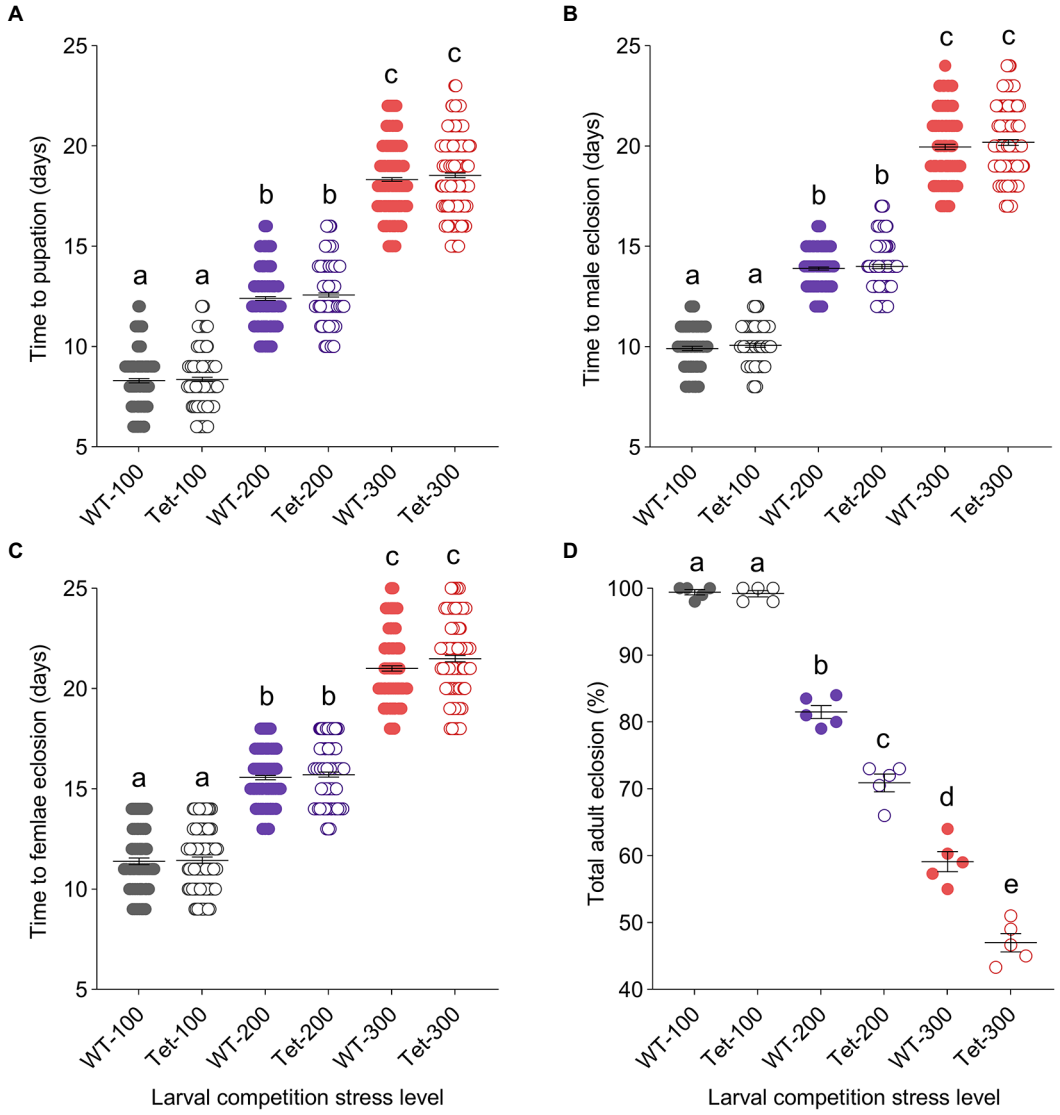
The effects of *Wolbachia* infection and competition stress on *Culex quinquefasciatus* development. Time to pupation was extended for *Cx. quinquefasciatus* larvae after exposure to increased levels of competition stress (Low: gray dots, 100 larvae per pan; Medium: purple dots, 200 larvae per pan; and High: red dots, 300 larvae per pan). However, no difference in mean pupation time was observed between WT (*Wolbachia*+, filled circles) and Tet (*Wolbachia*-, empty circles) larvae at any level of competition stress **(A)**. Similarly, the time that adult male mosquitoes **(B)** and adult female mosquitoes **(C)** took to eclose was extended under higher competition stress, but not impacted by *Wolbachia*. Adult eclosion rates **(D)** decreased when competition stress was increased, and at medium and high competition stress levels, *Wolbachia*-infected mosquitoes had a higher rate of eclosion than their uninfected counterpart lines. In panels **(A–C)**, each dot represents data from an individual mosquito. In panel **(D)**, each dot represents the percentage of adults that enclosed from a single pan. Horizontal lines in data sets represent treatment means ± s.e.m. Different lower-case letters above data sets indicate statistically significant differences between treatment groups (Tukey’s test; *p* < 0.05).

Larvae from the WT (*Wolbachia*+) and Tet (*Wolbachia*-) lines were reared independently under three distinct competition stress levels to assess whether *Wolbachia* infection status and larval competition stress level interact to impact development, fitness, *Wolbachia* density, and WNV infection in *Cx. quinquefasciatus* (Figure 1). Our data indicate that higher competition stress extended time to pupation (Figure 2A: Poisson Regression; *χ^2^* = 838.83, *df* = 2, *p* < 0.0001), time to eclosion for male mosquitoes (Figure 2B: Poisson Regression; *χ^2^* = 392.90, *df* = 2, *p* < 0.0001) and time to eclosion for female mosquitoes (Figure 2C: Poisson Regression; χ^2^ = 320.96, *df* = 2, *p* < 0.0001). However, none of these traits were affected by *Wolbachia* infection status (Poisson Regression; *p* > 0.05). Pairwise comparisons within competition treatments confirmed the lack of impact of *Wolbachia* infection on pupation or eclosion time at any competition level (Tukey’s test; *p* > 0.05).

Adult eclosion rate data (Figure 2D) showed significant effects associated with competition level (Logistic Regression; *χ^2^* = 244.55, *df* = 2, *p* < 0.0001), *Wolbachia* infection (Logistic Regression; *χ^2^* = 28.76, *df* = 1, *p* < 0.0001), and *Wolbachia* x competition interaction (Logistic Regression; *χ^2^* = 112.13, *df* = 2, *p* < 0.0001). Pairwise comparisons across treatment levels revealed no difference in eclosion rates between the WT and Tet lines at low competition (Tukey’s test; *p* = 0.9995), however, for the medium [WT eclosion rate = 81.5% ± 0.87, Tet eclosion rate = 70.9% ± 1.17 (average ± s.e.m.); Tukey’s test; *p* < 0.0001] and high competition stress treatments [WT eclosion rate = 59.1% ± 1.35, Tet eclosion rate = 47.0% ± 1.22 (average ± s.e.m.); Tukey’s test; *p* < 0.0001], a significantly greater proportion of WT mosquitoes eclosed compared to Tet mosquitoes, suggesting that *Wolbachia* infection has the potential to promote development and survival when larval competition stress is high.

### 3.2. Adult size and *Wolbachia* density

**Figure 3:**
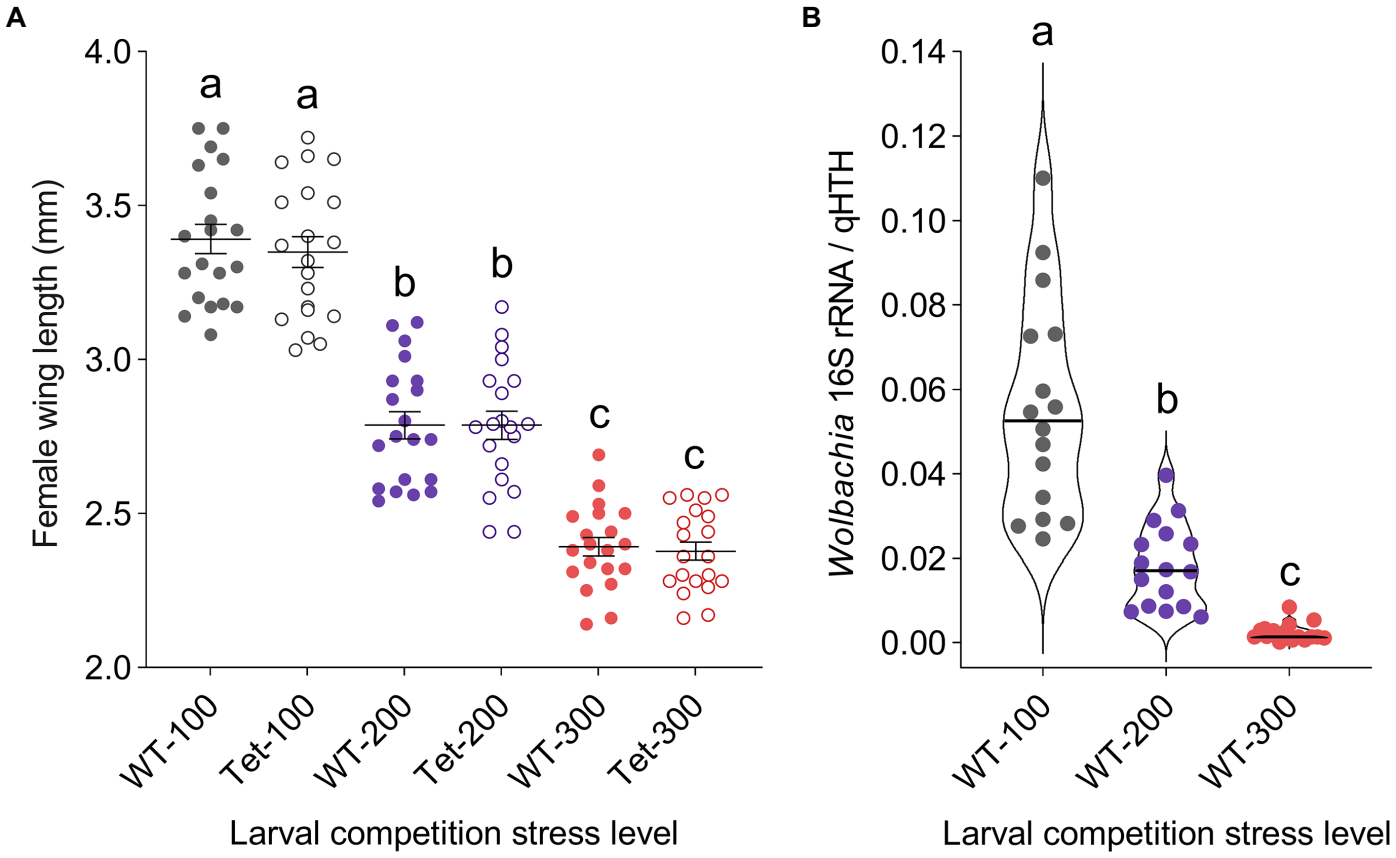
The effects of *Wolbachia* infection and competition stress on *Culex quinquefasciatus* size and *Wolbachia* density. Wing length was measured for WT and Tet adults reared under the three competition stress treatments as a proxy for body size **(A)**. Mosquitoes reared under higher competition stress had shorter wings, indicating a smaller body size. There was no impact of *Wolbachia* infection (Two-way ANOVA). Dots represent data from individual female mosquitoes, while horizontal lines indicate treatment means ± s.e.m. *Wolbachia* density was quantified for the three WT lines using qPCR, comparing copies of the *Wolbachia* 16 s rRNA gene relative to the host homothorax gene (qHTH; **B**), with significantly lower density associated with increasing competition stress (Kruskal-Wallis test; *p* < 0.0001). Violin plots in **(B)** highlight the distribution of *Wolbachia* density data, with dots representing single samples and horizontal lines representing treatment medians. Different lower-case letters above data sets indicate statistically significant differences between treatment groups.

Adult female size (Figure 3A) estimated *via* wing length was significantly decreased by higher larval competition (Two-way ANOVA; *F* = 277.12, *df* = 2, *p* < 0.0001), but was not affected by *Wolbachia* infection (Two-way ANOVA; *F* = 0.31, *df* = 1, *p* = 0.5811) or by the interaction of those two variables (Two-way ANOVA; *F* = 0.13, *df* = 2, *p* = 0.8765). *Wolbachia* density (Figure 3B) also decreased as competition stress increased (Kruskal-Wallis ANOVA; *χ^2^* = 39.27, *df* = 2, *p* < 0.0001), with significant differences in density observed between each of the three competition stress treatments. On average, we observed that *Wolbachia* density decreased by 67.39% between the low and medium competition stress treatments, and by 95.68% between the low and high competition stress treatments (Dwass-Steel-Critchlow-Fligner test; low vs medium—*p* < 0.0001: low vs high—*p* < 0.0001: medium vs high—*p* < 0.0001).

### 3.3. Prevalence of West Nile virus infection

**Figure 4:**
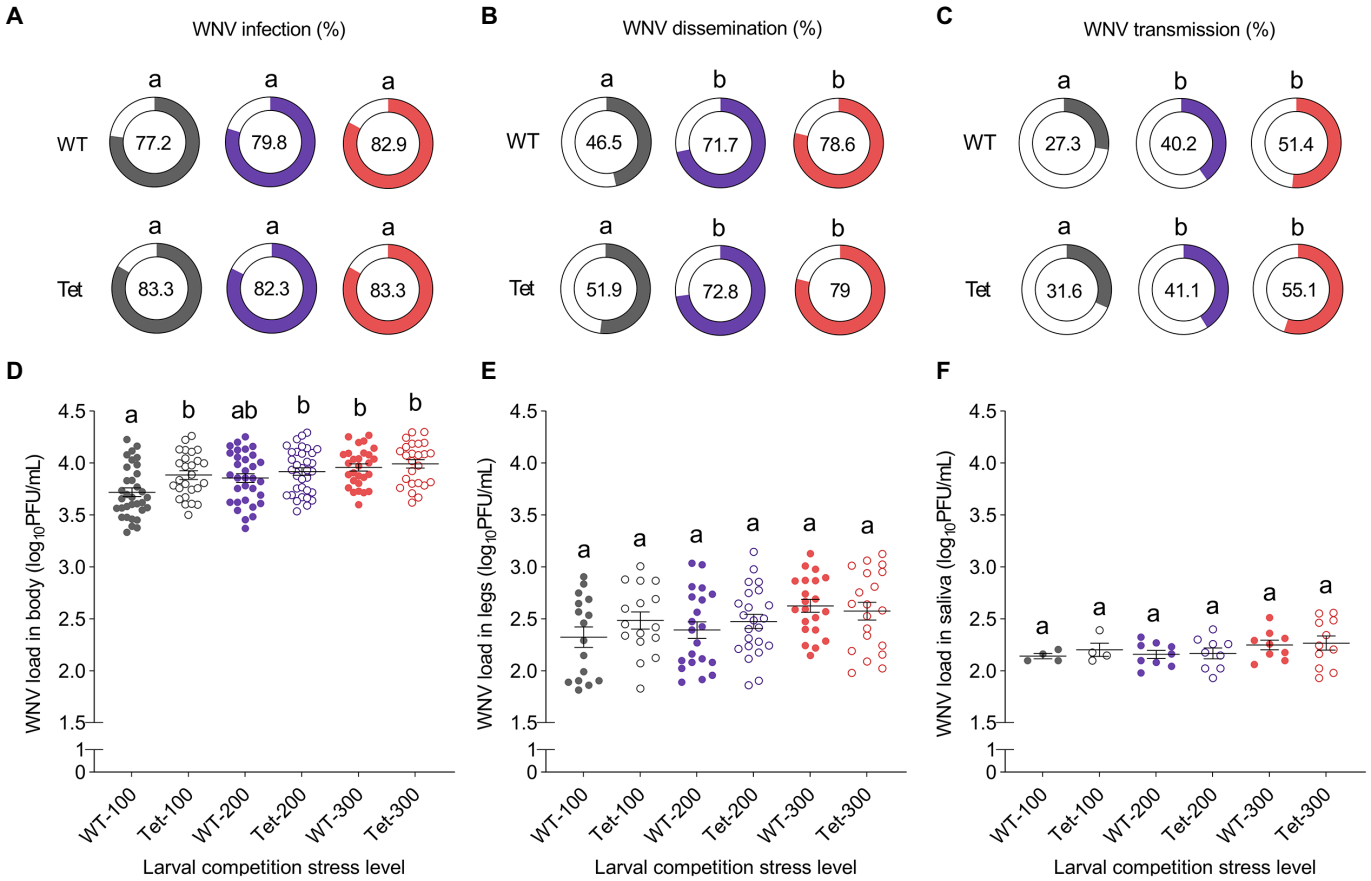
The impact of *Wolbachia* infection and larval competition on WNV infection in *Culex quinquefasciatus*. Prevalence of WNV infection was measured in the bodies **(A)**, legs **(B)**, and saliva **(C)** of female *Cx. quinquefasciatus* mosquitoes at 14 days post-oral challenge. Significant increases in the prevalence of infection were associated with increased competition stress in legs (dissemination), and saliva (transmission) specimens, but not in mosquito bodies (Logistic Regression: *p* < 0.05). Filled areas on donut charts and central numbers represent the percentage of specimens positive for WNV *via* plaque-forming assay. WNV load in bodies **(D)**, legs **(E)**, and saliva **(F)** was also determined *via* plaque-forming assay. Overall, increased competition stress led to increased WNV load in mosquito bodies and legs (Two-way ANOVA; *p* < 0.05). However, *Wolbachia* infection reduced WNV loads in mosquito bodies at low and medium competition stress treatments (Two-way ANOVA: *p* < 0.01). No effects of competition or *Wolbachia* were seen in saliva samples. Dots represent WNV load values from individual mosquito tissues/saliva samples. Horizontal lines indicate treatment means ± s.e.m. Different lower-case letters above data sets indicate statistically significant differences between treatment groups determined *via* pairwise comparisons.

The prevalence of WNV infection in mosquito tissues was determined in mosquito bodies (body infection rate), legs (dissemination rate), and saliva (transmission rate) at 14 dpi *via* plaque-forming assay. We observed no effects of any of our test variables on WNV body infection rates (Figure 4A) with viral prevalence observed to be between 77 and 84% for all treatments (Logistic Regression; Competition: *χ^2^* = 0.07, *df* = 2, *p* = 0.9642; *Wolbachia* infection: *χ^2^* = 0.21, *df* = 1, *p* = 0.6442; Interaction *χ^2^* = 0.07, *df* = 2, *p* = 0.9643). Dissemination rates (Figure 4B) were significantly impacted by competition stress (Logistic Regression; *χ^2^* = 7.85, *df* = 2, *p* = 0.0197), with higher infection rates associated with the medium and high competition stress treatments. However, there were no significant effects due to *Wolbachia* infection (Logistic Regression; *χ^2^* = 0.14, *df* = 1, *p* = 0.7010), or *Wolbachia* infection × competition interaction (Logistic Regression; *χ^2^* = 0.56, *df* = 2, *p* = 0.7553). Similar effects were observed with WNV transmission rates (Figure 4C), where higher competition stress increased the prevalence of infection (Logistic Regression; *χ^2^* = 8.79, *df* = 2, *p* = 0.0123), but neither *Wolbachia* infection (Logistic Regression; *χ^2^* = 0.35, *df* = 1, *p* = 0.5534) nor the interaction term (Logistic Regression; *χ^2^* = 0.10, *df* = 2, *p* = 0.9483) had an effect.

### 3.4. West Nile virus load

West Nile virus load was quantified in all body, leg, and saliva samples determined to be positive for WNV infection. WNV load in body samples (Figure 4D) increased as larval competition increased (Two-way ANOVA; *F* = 8.68, *df* = 2, *p* = 0.0003). We also observed a significant effect of *Wolbachia* infection (Two-way ANOVA; *F* = 7.04, *df* = 1, *p* = 0.0087), with lower WNV loads linked to *Wolbachia* infection in the low and medium competition stress treatments, although only the former was significant *via* pairwise comparisons. No significant effects of competition by *Wolbachia* interaction were observed for this trait (Two-way ANOVA; *F* = 1.40, *df* = 2, *p* = 0.2505). In contrast, WNV load in mosquito legs (Figure 4E) was significantly influenced by competition treatment (Two-way ANOVA; *F* = 3.59, *df* = 2, *p* = 0.0308), whereas *Wolbachia* infection (Two-way ANOVA; *F* = 0.98, *df* = 1, *p* = 0.3245) and its interaction with competition (Two-way ANOVA; *F* = 0.88, *df* = 2, *p* = 0.4187) did not have significant effects. For this trait, higher competition stress led to a general increase in WNV load. For WNV transmission (Figure 4F), we observed no significant effects of competition (Two-way ANOVA; *F* = 1.83, *df* = 2, *p* = 0.1728), *Wolbachia* infection (Two-way ANOVA; *F* = 0.34, *df* = 1, *p* = 0.5610), or their interaction (Two-way ANOVA; *F* = 0.08, *df* = 2, *p* = 0.9263) on WNV load in mosquito saliva.

## 4. Discussion

Our data highlight a fitness-associated protective effect associated with native *Wolbachia* infection in *Cx. quinquefasciatus* mosquitoes with potentially interesting implications for WNV infection and transmission in nature. We observed that high competition stress had strong impacts on mosquito biology and WNV infection, regardless of *Wolbachia* infection status. We saw that high competition stress extended development time, decreased the likelihood of eclosion, decreased female body size, and increased susceptibility to WNV infection. However, when mosquito larvae were exposed to medium or high levels of competition stress, we observed that *Wolbachia*-infected mosquitoes experienced a significantly lower rate of mortality than their uninfected counterparts. Our WNV infection data highlight increased prevalence and WNV load for mosquitoes reared under high competition stress, as well as a moderate decrease in WNV load associated with *Wolbachia* infection that occurred only when competition stress was lower. Consequently, our data suggest that under low competition stress, native *Wolbachia* infection in *Cx. quinquefasciatus* potentially offers a low degree of blocking of WNV infection; however, under high competition stress, *Wolbachia* promotes the survival of mosquitoes and does not restrict WNV infection.

### 4.1. Effects of competition stress

Competition during development is a critical factor that impacts mosquito population dynamics by influencing key fitness traits such as longevity and body size, with both of these traits strongly linked to vector competence (Moore and Fisher, 1969; Gilles et al., 2011; Walsh et al., 2011). Our results were consistent with previous studies of competition stress in mosquitoes. We observed that the development time of *Cx. quinquefasciatus* was extended after larvae were reared under high competition stress where the availability of food and space was limited. We also observed a strong negative correlation between competition stress and adult size. These findings are consistent with previous reports that demonstrate that high competition increases mosquito development time and decreases adult body size (Wada, 1965; Agnew et al., 2000; Mpho et al., 2000; Costanzo et al., 2005; Alto et al., 2008, 2015; Roberts and Kokkinn, 2010; Bara et al., 2015).

Our observation that high competition stress increased susceptibility to WNV, following midgut infection, mirrors findings from previous studies, which highlight links between competition stress and arboviral infection (Alto and Lounibos, 2013; Kim and Muturi, 2013). Smaller mosquitoes, resulting from high competition, were more susceptible to infection with DENV or Sindbis virus, experiencing higher rates of body and dissemination than those reared under low competition stress (Alto et al., 2005, 2008). Similarly, *Ae. aegypti* mosquitoes that experienced competition stress had a thinner midgut basal lamina and exhibited increased susceptibility to ZIKV infection (Herd et al., 2021). However, these interactions can vary depending on the host-pathogen combination. For instance, some studies have observed a positive relationship between mosquito body size and viral infection rates, as for *Aedes triseriatus* and La Crosse virus (Bevins, 2008). However, these larger sized mosquitoes were from nutrient deprived conditions (high competition stress) which enhanced larval mortality, and comparatively increased nutrient availability for the few survivors. Others have found no relationship between these traits, as with *Cx. tarsalis* and WNV (Dodson et al., 2011).

### 4.2. Protective impact of *Wolbachia* infection during competition stress

Competition stress is a major cause of mortality for immature mosquitoes (Alto et al., 2005, 2008; Reiskind and Lounibos, 2009; Alto and Lounibos, 2013). In our experimental design, we observed that higher competition stress significantly increased mosquito mortality among juvenile mosquitoes, with a mortality rate of approximately 50 % observed in the high competition treatment. Interestingly, we observed a protective effect associated with *Wolbachia* that reduced the rate of mortality seen in the medium and high competition stress treatments. As such, our data indicate that *w*Pip infection offers a fitness advantage to our mosquito colony during sub-optimal developmental conditions. These findings differ from observations on native *Wolbachia* infections in *Ae. albopictus* where eclosion rates under competition stress were similar between *Wolbachia*-infected and uninfected mosquitoes (Gavotte et al., 2014). In another study utilizing an *Ae. albopictus* population of mixed *Wolbachia* infection status, *Wolbachia* actually induced a fitness cost by reducing the adult eclosion rate under high competition stress (Gavotte et al., 2010). It is currently unclear if similar effects occur with other *Cx. quinquefasciatus* populations, other *w*Pip genetic variants, or in other *Wolbachia* strain-mosquito combinations.

While we saw a positive relationship between competition stress level and development time and a negative relationship between competition stress level and adult size, we did not see an effect of *Wolbachia* infection on either of those two traits. These findings are consistent with a previous study examining competition and the native *Wolbachia* infections of *Ae. albopictus*, which also observed no significant impacts on development time or adult size (Islam and Dobson, 2006). A further study saw no effect of competition stress on female *Ae. albopictus* development but did observe a delay in development time for males (Gavotte et al., 2014).

Previous studies indicate that the fitness effects associated with *w*Pip in *Culex pipiens* complex mosquitoes are quite variable. For instance, in one study on *Cx. quinquefasciatus*, *w*Pip infection was associated with quicker larval development, a longer lifespan, and quicker egg development post-blood feeding, but *Wolbachia*-free mosquitoes laid more eggs and produced more viable progeny (de Almeida et al., 2011). In other studies, fecundity and fertility were similar for *Cx. pipiens* and *Cx. quinquefasciatus* regardless of the presence of *w*Pip (Rasgon and Scott, 2003; Díaz-Nieto et al., 2021). The *w*Pip strain has many different genetic variants, which display a complex pattern of CI phenotypes during crosses (Dumas et al., 2013; Altinli et al., 2018; Bonneau et al., 2018), and variation in symbiont genetics might contribute to some of these differential fitness effects. For instance, at least one *w*Pip variant decreases host susceptibility to the insecticidal bacterium *Bacillus thuringiensis* subsp. *israelensis*, while others have no impact (Díaz-Nieto et al., 2021).

Fitness effects associated with development and competition appear to differ between transinfections and native *Wolbachia* infections, in line with the hypothesis that transinfections, representing more novel host-symbiont relationships, lead to more extreme fitness consequences for the host (Zug and Hammerstein, 2015a). For instance, *w*MelPop, a virulent, life-shortening *Wolbachia* strain, extends *Ae. aegypti* development time when nutritional stress is low and crowding stress is high (Yeap et al., 2011), while crowding-induced competition, *w*MelPop infection, and their interaction all significantly reduced *Ae. aegypti* survival rates (Suh et al., 2017). In *Ae. aegypti* transinfected with the less virulent *w*Mel strain, high nutritional stress leads to changes in wing shape (Yeap et al., 2013), reduces body size (Yeap et al., 2013; Dutra et al., 2016), and reduces development time (Dutra et al., 2016). In a further study, three different *Wolbachia* strains, *w*Mel, *w*MelPop, and *w*AlbB, all reduced *Ae. aegypti* survival rate under extreme starvation conditions (Ross et al., 2016).

### 4.3. Potential consequences for WNV transmission in nature

We observed a modest decrease in WNV load associated with *Wolbachia* infection in mosquitoes reared under low competition stress. Without an accompanying reduction in overall susceptibility or transmission rate, it is difficult to infer that a similar effect would directly impact WNV transmission in mosquitoes in nature. However, taken in the context of our observations that *Wolbachia* infection promoted mosquito survival under high competition conditions, and that mosquitoes exposed to higher competition stress were generally more susceptible to WNV infection, our results reveal some interesting insights into the potential modulatory role of native *Wolbachia* infections on mosquito-arbovirus interactions in nature. In such a system, *Wolbachia* infection could promote the survival of mosquitoes that are potential vectors of WNV. As such, we consider it vital to evaluate whether similar effects occur in other mosquito populations that naturally harbor *Wolbachia* as competition stress and incomplete penetrance of native *Wolbachia* infections in certain populations could potentially be impacting vectorial capacity.

We also observed that *Wolbachia*-infected *Cx. quinquefasciatus* exposed to high competition stress as larvae experienced reduced adult size and reduced *Wolbachia* density. As established above, smaller body size in mosquitoes can lead to increased susceptibility to arboviral infection (Alto et al., 2015). Previous studies have demonstrated that crowding stress experienced by *Ae. albopictus* larvae leads to reduced density of *w*AlbA and *w*AlbB in adult mosquitoes (Wiwatanaratanabutr and Kittayapong, 2009). Increased nutritional stress leads to reduced *Wolbachia* density in adult mosquitoes (Dutton and Sinkins, 2004). There are also strong links between nutrient availability and *Wolbachia* density (Ponton et al., 2015; Caragata et al., 2016), and between *Wolbachia* density and pathogen blocking (Walker et al., 2011; Joubert et al., 2016). As such, it is possible that the loss of the minor WNV blocking phenotype we observed at low competition stress was, at least in part, driven by a loss of *Wolbachia* density, and that decrease in density was driven by decreased nutrient availability. Previous studies have linked between-population variation in *Wolbachia* density (Micieli and Glaser, 2014) and seasonality-driven changes in *Wolbachia* density (Novakova et al., 2017) to differences in the ability of *Wolbachia* to block WNV infection in *Cx. quinquefasciatus*. Taken together with our data, these findings highlight the great potential for environmental change to drive variation in interactions between arboviruses and mosquitoes with a native *Wolbachia* infection.

### 4.4. Study caveats and future directions

A major caveat in this study is that experiments were performed with a single population of *Cx. quinquefasciatus* mosquitoes and a single WNV isolate. Given the breadth of host-symbiont-pathogen interactions seen with native *Wolbachia* infections, it is possible that repeating the study with a different mosquito genotype, a different mosquito species, or a different pathogen could have produced different results (e.g., host-symbiont-pathogen interactions). Performing such studies across different mosquito-pathogen-*Wolbachia* strain combinations is essential to understand the extent to which native *Wolbachia* infections modulate the vector competence of mosquito populations. We could potentially have observed differential results if these assays had been replicated under field conditions, or in mosquito lines with field microbiomes, given the increased diversity of field microbiomes in mosquitoes compared to laboratory microbiomes. The inclusion of a field-derived microbiome could provide a scope to influence and modulate host-pathogen interactions, and host-pathogen-*Wolbachia* interactions. Additionally, impacts of crowding stress on the microbiome and *Wolbachia*-microbiome interactions have not been explored in *Cx. quinquefasciatus* mosquitoes, and such interactions could have contributed to the results of our fitness and vector competence assays. Similarly, it is possible that our results were impacted by our decision to utilize both nutritional and crowding stress as part of our competition stress conditions, which meant that there was variable access to food per larva across larval density treatments. Future studies should consider examining how independent and combined effects of varying nutrient availability and larval density impact interactions between *Wolbachia*, mosquitoes and arboviruses.

Lingering impacts of antibiotic treatment or the inability to re-constitute key members of the microbiome in the Tet line could have influenced traits associated with fitness or vector competence, particularly if this included microorganisms that were highly responsive to nutrient availability and competition stress. Another potential caveat is the low number of WNV-positive saliva samples in our transmission assay (*N* = 4–11 per treatment), which potentially limited our ability to detect significant effects associated with *Wolbachia* infection or competition stress on that trait. Finally, an important point to note is that, given the inherent differences in the nature of the host-symbiont-pathogen interactions and the differing response to competition stress discussed above, our findings here are unlikely to have direct relevance to the biology the *Wolbachia*-transinfected mosquitoes being utilized in mosquito control interventions, or on the ability of those *Wolbachia* strains to induce pathogen blocking.

## 5. Conclusion

Our data demonstrate the importance of environmental stresses as modulators of mosquito-*Wolbachia*-pathogen relationships in mosquitoes that naturally harbor *Wolbachia*. Here we demonstrate that larval competition stress in *Cx. quinquefasciatus* strongly modulates larval development, adult eclosion rate, adult size, and WNV vector competence. Our results also show that, under high competition stress, *Wolbachia* infection confers a fitness advantage to its host, increasing the likelihood of survival to eclosion. However, these surviving mosquitoes are smaller, have reduced *Wolbachia* density, and show increased susceptibility to WNV compared to mosquitoes reared under low competition stress conditions. As such, the combination of native *Wolbachia* infection and high competition stress, either through reduced nutrient availability or increased crowding, could be having unexpected consequences on vector competence in mosquito populations in nature.

## Data availability statement

The raw data supporting the conclusions of this article will be made available by the authors, without undue reservation.

## Ethics statement

The animal study was reviewed and approved by University of Florida Institutional Biosafety Committee and Biohazard Project Registrations and Institute of Animal Care and Use Committee.

## Author contributions

AA, BA, and EC contributed to conception and design of the study, performed the statistical analysis, and reviewed and edited the manuscript and generated the final version of the manuscript. AA, DP-R, DK, NK, and BE performed the experiments. AA wrote the first draft of the manuscript. All authors contributed to the article and approved the submitted version.

## Funding

This research was supported by the United States Department of Agriculture (USDA) and National Institute of Food and Agriculture (NIFA), Hatch project (1026692) to EC.

## Conflict of interest

The authors declare that the research was conducted in the absence of any commercial or financial relationships that could be construed as a potential conflict of interest.

## Publisher’s note

All claims expressed in this article are solely those of the authors and do not necessarily represent those of their affiliated organizations, or those of the publisher, the editors and the reviewers. Any product that may be evaluated in this article, or claim that may be made by its manufacturer, is not guaranteed or endorsed by the publisher.
